# Supplementary material for: Using Hormone Data and Age to Pinpoint Cycle Day within the Menstrual Cycle
Source: Medicina (Kaunas). 2023 Jul 23;59(7):1348. doi: 10.3390/medicina59071348 (PMC10384168; doi:10.3390/medicina59071348)
Supplement: Supplementary file 1 [file medicina-59-01348-s001.zip › Unlocking the Mysteries of Menstrual Cycles - Supplementary Material v3.pdf]

Supplementary Materials

Age Range (years)	BMI (lb/in^2)	Total Cycles Monitored	Race	n
25-29	26.14	624	White	180
			Asian	20
			Black or African American	13
			Other	18
30-34	24.43	1766	White	450
			Asian	36
			Black or African American	16
			Other	43
35-39	27.21	1266	White	253
			Asian	33
			Black or African American	13
			Other	46
40-44	25.18	467	White	78
			Asian	12
			Black or African American	12
			Other	10

Supplementary Table S1: Demographic data of Oova users included in the study. Age and race data were self-reported, as were weight and height, which were used to calculate BMI.

Age Range (years)	n (Follicular Phase)	n (Luteal Phase)	p-value
25-29	211	276	1.63×10^{-23}
30-34	772	1031	4.18×10^{-40}
35-39	557	739	3.89×10^{-9}
40-44	203	272	3.52×10^{-3}

Supplementary Table S2: Significance results for cycle phase lengths between age groups. Results from the t-test highlight the significance between follicular and luteal phase lengths in each age group.

Follicular Phase Length				
**Age Range 1** (years)	**Age Range 2** (years)	**n** (Age Range 1)	**n** (Age Range 2)	**P-value**
25-29	30-34	211	722	2.9×10^{-2}
25-29	35-39	211	557	4.3×10^{-6}
25-29	40-44	211	203	1.5×10^{-8}
30-34	35-39	722	557	8.6×10^{-5}
30-34	40-44	722	203	3.1×10^{-7}
35-39	40-44	557	203	1.7×10^{-2}

Supplementary Table S3: Pairwise comparison of follicular phase length between age groups. Two-sided t-tests were performed to assess the significance of differences in follicular phase length across all age groups. The associated p-values are presented.

Luteal Phase Length				
Age Range 1 (years)	Age Range 2 (years)	n (Age Range 1)	n (Age Range 2)	P-value
25-29	30-34	276	1031	4.7×10^{-2}
25-29	35-39	276	739	2.6×10^{-4}
25-29	40-44	276	272	3.2×10^{-2}
30-34	35-39	1031	739	1.5×10^{-2}
30-34	40-44	1031	272	0.47569
35-39	40-44	739	272	0.27851

Supplementary Table S4: Pairwise comparison of luteal phase length between age groups. Two-sided t-tests were performed to assess the significance of differences in luteal phase length across all age groups. The associated p-values are presented.

	Follicular			Luteal		
Cycle Day	n	LH (mIU/L)	PdG (ng/mL)	n	LH (mIU/L)	PdG (ng/mL)
**10**	100	5.31 ± 1.22	2.68 ± 0.40			
**11**	115	3.71 ± 0.54	2.64 ± 0.27			
**12**	142	5.23 ± 0.99	3.31 ± 0.37			
**13**	132	5.00 ± 0.57	2.69 ± 0.30			
**14**	124	5.83 ± 1.17	3.19 ± 0.39			
**15**	113	6.46 ± 1.31	3.28 ± 0.42			
**17**				101	7.82 ± 1.00	8.52 ± 0.74
**18**				116	7.19 ± 0.86	8.86 ± 0.68
**19**				119	6.73 ± 1.09	9.10 ± 0.69
**20**				127	6.71 ± 0.73	11.08 ± 0.70
**21**				121	5.58 ± 0.73	10.54 ± 0.69
**22**				121	4.69 ± 0.62	11.34 ± 0.66
**23**				119	4.21 ± 0.52	10.97 ± 0.57

Supplementary Table S5: The average LH and PdG levels for each day of the cycle for the 25-29 age group. This table only includes data points with at least 100 samples. Any data point with less than 100 samples was excluded to prevent any potential misinterpretation.

	Follicular			Luteal		
Cycle Day	n	LH (mIU/L)	PdG (ng/mL)	n	LH (mIU/L)	PdG (ng/mL)
9	205	4.26 ± 0.47	2.13 ± 0.19			
10	325	3.94 ± 0.34	2.19 ± 0.15			
11	371	5.45 ± 0.48	2.09 ± 0.14			

12	388	5.90 ± 0.45	2.20 ± 0.16			
13	349	6.99 ± 0.57	2.10 ± 0.14	121	12.99 ± 1.15	5.83 ± 0.50
14	291	6.91 ± 0.60	2.37 ± 0.18	197	12.33 ± 0.94	6.56 ± 0.46
15	224	7.19 ± 0.69	2.25 ± 0.20	264	11.14 ± 0.92	7.39 ± 0.40
16	176	8.00 ± 0.83	2.56 ± 0.26	310	9.33 ± 0.68	8.34 ± 0.41
17	126	9.15 ± 1.34	3.12 ± 0.38	348	7.50 ± 0.53	8.98 ± 0.39
18				382	6.93 ± 0.50	10.07 ± 0.38
19				378	6.03 ± 0.42	10.45 ± 0.39
20				388	4.70 ± 0.37	11.58 ± 0.38
21				367	4.18 ± 0.39	11.84 ± 0.40
22				350	3.67 ± 0.33	12.02 ± 0.40
23				309	4.20 ± 0.44	11.22 ± 0.44
24				231	4.56 ± 0.69	12.36 ± 0.51
25				160	4.30 ± 0.61	11.30 ± 0.61
26				115	4.79 ± 0.76	11.33 ± 0.72

Supplementary Table S6: The average LH and PdG levels for each day of the cycle for the 35-39 age group. This table only includes data points with at least 100 samples. Any data point with less than 100 samples was excluded to prevent any potential misinterpretation.

	Follicular			Luteal		
**Cycle Day**	**n**	**LH** (mIU/L)	**PdG** (ng/mL)	**n**	**LH** (mIU/L)	**PdG** (ng/mL)
10	128	4.34 ± 0.64	1.71 ± 0.21			
11	156	5.58 ± 0.72	1.71 ± 0.19			
12	146	7.18 ± 0.79	1.77 ± 0.19			
13	120	8.97 ± 1.14	2.09 ± 0.28			
15				121	12.03 ± 0.99	7.51 ± 0.63
16				144	9.08 ± 0.88	8.05 ± 0.56
17				143	8.57 ± 0.90	9.38 ± 0.58
18				143	5.75 ± 0.67	11.30 ± 0.62
19				144	5.32 ± 0.64	12.02 ± 0.61
20				140	4.91 ± 0.69	13.55 ± 0.61
21				123	4.77 ± 0.65	12.34 ± 0.65
22				125	5.15 ± 0.65	12.31 ± 0.63
23				115	4.46 ± 0.64	12.18 ± 0.70

Supplementary Table S7: The average LH and PdG levels for each day of the cycle for the 40-44 age group. This table only includes data points with at least 100 samples. Any data point with less than 100 samples was excluded to prevent any potential misinterpretation.
